# Student satisfaction with videoconferencing teaching quality during the COVID-19 pandemic

**DOI:** 10.1186/s12909-020-02310-2

**Published:** 2020-10-31

**Authors:** Tarah H. Fatani

**Affiliations:** Department of Pediatrics, Pediatric Endocrinology, King Abdulaziz University, Jeddah, 21589 Saudi Arabia

**Keywords:** Student satisfaction, Student evaluation of education quality, Community of inquiry, Videoconferencing, COVID-19

## Abstract

**Background:**

The coronavirus disease 2019 pandemic prompted the pediatric department at King Abdulaziz University to continue students’ educational activities by offering courses online that utilized web video conferencing (WVC). Given the uncertainties of WVC educational quality and the challenge of shifting to an online environment, this study aimed to evaluate student satisfaction with the teaching quality of case-based discussion (CBD) sessions conducted through WVC.

**Methods:**

One hundred sixty-two undergraduate medical students in pediatrics completed the reduced Students’ Evaluation of Educational Quality (SEEQ) survey with a five-point Likert scale over 5 weeks. The WVC CBD sessions were facilitated by 50 faculty members.

**Results:**

82% of respondents were highly satisfied with the WVC CBD session’s teaching quality. The majority agreed that the sessions were intellectually challenging, that the instructors were dynamic, and encouraged students to participate. No statistically significant correlation was found between student satisfaction and technical issues (*r* = 0.037*, p* = 0.003).

**Conclusions:**

WVC teaching had an overall positive outcome on student satisfaction, and teaching quality relied on teaching, cognitive, and social presence rather than technology. However, technology remains an important platform that supports teachers’ educational activities. Thus, implementing a blended pediatric course to augment future course delivery is optimal.

## Background

The coronavirus disease 2019 (COVID-19) pandemic has changed medical education; the United Nations Educational, Scientific, and Cultural Organization (UNESCO) announced that 1.2 billion students across the planet have been affected by school and university closures due to the pandemic [1]. Saudi Arabia is one of the many countries that made the unprecedented decision to close schools and universities to contain the spread of the deadly virus as social distancing has been the most effective preventative strategy for COVID-19 pending development of a vaccine, treatment, or both [2].

At King Abdulaziz University, this closure dramatically interrupted medical students’ educational activities and clinical training, prompting the institution to move the course of study online and contemplate new approaches to distance education. Faculty members in the pediatric department shifted from in-person to distance education to facilitate students’ education. However, for many in the department, web video conferencing (WVC) technologies and online education were new and challenging.

The educators had to meet four major online education challenges: demonstrating pedagogical skills in an online classroom, addressing their managerial role, establishing relationships with students, and providing technical support [3, 4]. Despite the challenges, the instructors had to effectively facilitate case-based discussion (CBD) via WVC while maintaining a high-quality, authentic learning experience with high student satisfaction rates.

### Distance education and student satisfaction

Distance education is defined as learning activities within formal, informal, or non-formal domains that are facilitated by information and communication technologies to lessen distance, both physically and psychologically, and to increase interactivity and communication among learners, learning sources, and facilitators [5]. Distance education has become the basic global mode of course delivery, and the quality of this delivery is essential.

The five elements in the Sloan quality framework for effective online education are student satisfaction, learning effectiveness, faculty satisfaction, student access, and institutional cost-effectiveness [6]. Furthermore, the root causes of online learning success are motive and leadership, focus on the program, faculty support, students (satisfaction, services, outcome), and growth in student enrolment [7]. Thus, the quality of online learning is highly influenced by teaching rather than technology [7].

A systematic review of randomized controlled trials concluded that digital problem-based learning (DPBL) was potentially more effective in improving knowledge and skills than traditional problem-based learning (PBL) due to greater student interaction and engagement in DPBL [8]. However, the review found mixed data concerning student satisfaction outcomes [8]. Various studies on student satisfaction with online learning have identified the following factors: instructor interaction, communication, active learning, students’ ability to initiate and control their actions in the learning environment, efficient assessment of academic progress, technology, and the learning environment [9, 10].

### Video conferencing and student satisfaction

WVC is a synchronous model for interactive voice, video, and data transfer between two or more groups. It enables real-time, two-way video and audio communication as well as content sharing and messaging between instructors and students [11, 12]. Additionally, it allows immediate feedback and supports collaborative learning among students [11, 12]. WVC allows learners and instructors to participate in web-based discussions that are not bound to certain hardware or software, utilizing services such as Zoom, Skype for Business, and GoToMeeting [12]. As technology advances, WVC has become one of the most common tools used for synchronous online teaching [13, 14]. It promotes dynamic collaborative efforts, although it does face challenges with technical issues [12].

The findings on student satisfaction with WVC have been mixed. Dawson showed that synchronous communication increases the sense of classroom community and, therefore, student satisfaction [15]. Additionally, in a study by Dogget, over 90% of students responded favourably to the instructor’s use of WVC and his/her encouragement to ask questions; however, 80% stated that they would have been more comfortable in a conventional classroom setting, and 57% agreed that WVC technology was a barrier to interacting with the instructor [16]. Smith et al. [17] demonstrated that WVC was an effective method for satisfactorily teaching surgical tutorials to medical students. In contrast, Giesbers et al. [18] compared the learning experience of students using discussion forums (DF) versus students using both DF and WVC (which is expected to increase social presence) and found that WVC was not associated with enhanced student learning during their four-year experience with WVC. The only exceptions were the clarification of the module’s goals and tasks [19].

### Video conferencing and case-based discussions

CBD is an interactive, student-centered learning approach closely related to PBL. CBD utilizes real-life scenarios that the students would have faced during the clinical phase of their pediatric rotation [18]. It aims to enable students to learn from patients’ cases, to enhance their problem-solving skills, and to encourage them to think analytically about the patient’s case from various angles. CBD has several advantages. It promotes active, self-directed, life-long learning; integrates basic medical knowledge with clinical cases; emphasizes clinical reasoning when approaching a case; and develops student communication skills [18]. Information about the case from history, physical examination, and investigation is given in a step-wise manner to allow students to analyze the information and generate differential diagnoses. The students then strive to reach the most likely diagnosis and appropriate management.

WVC is an optimal choice to facilitate CBD sessions as it will increase social presence which help the acquisition of information, focus on learner-centered opportunities, engage students and allow small group interactions, allow instructors to observe and assess students working in real-life learning experience, develop positive communication skills, demonstrate leadership, and shared responsibility as they analytically think about how to approach a Pediatric case. However, problems using WVC for conducting the sessions are destined to occur such as experiencing technology issues, confidence navigating the platform by both instructor and students, ensuring student engagement, and delivering creative and effective educational practices in an online learning environment [20].

### Community of inquiry framework

The community of inquiry (COI) framework constitutes three critical elements of online education—cognitive, teaching, and social presence—as well as the internal dynamic relationships among them [21]. Presence is defined as a state of alert awareness, receptivity, and connectedness to the mental, emotional, and physical workings of the individual and the group in the context of their learning environments, as well as the ability to respond with a considered and compassionate best next step [22]. Cognitive presence is the extent to which leaners are able to construct and confirm meaning through sustained reflection and discourse. It is implemented through the Practical Inquiry model consisting of four phases: triggering event, exploration, integration and resolution [21]. Teaching presence is the design, facilitation, and direction of cognitive and social processes, while social presence is the ability of the learners to identify with the community, communicate purposefully and develop inter-personal relationships by way of projecting their individual personalities [21]. These three interdependent elements are essential for delivering a successful asynchronous (i.e. not simultaneous) online course and creating an effective educational experience [21]. The SEEQ questionnaire shares the same concepts as the COI framework (cognitive, teaching, and social presence); questions exploring student learning reflect cognitive presence, questions exploring enthusiasm and organization reflect teaching presence, and questions on group interaction and individual rapport reflect teaching and social presence combined.

The relevance of the COI framework to synchronous (i.e., simultaneous interaction) online learning with WVC technology needs to be further verified. According to Moore, using interactive teleconference media offers the opportunity for dynamic inter-learner dialogue and student engagement, thereby enabling instructors to bridge the psychological and communication distance between instructors and learners and among learners. It also provides the opportunity to develop the cognitive skills of analysis and autonomy [23].

Given the novel exposure to distance education for both students and faculty members, this study aimed to evaluate student satisfaction with the teaching quality of case-based discussion (CBD) sessions conducted through WVC. Furthermore, students’ ongoing feedback about their satisfaction with the teaching quality will substantially impact future curriculum planning and design as further online pediatric courses are implemented.

## Methods

### Participants

This study enrolled 162 fifth-year male Saudi undergraduate medical students who were in the pediatric rotation course at King Abdulaziz University for 12 weeks from March to May 2020. The students were divided into 14 groups with 10–12 students in each group. All students were between 21 and 25 years old. The students’ attendance at each CBD was compulsory and monitored throughout the course.

### Pediatric course description

The pediatric course at King Abdulaziz University used the online platform Blackboard Ultra before the COVID-19 outbreak; this platform integrates course learning outcomes, student study guides, assignments, and assessments. Traditionally, the pediatric course is a 12-week rotation at the end of the academic year during which fifth-year medical students are exposed to clinical inpatient and outpatient environments and participate in face-to-face CBD and clinical sessions in a hospital. None of these educational activities was provided via distance education before the COVID-19 outbreak, and the CBD sessions were conducted by the students and facilitated by the teachers to enhance active learning. In order to prepare the faculty members for successful online course teaching, King Abdulaziz university provided faculty members with nonmandatory Blackboard development courses on online education and teaching, course delivery and navigation, and technical requirements.

The COVID-19 outbreak occurred 2 weeks through the rotation, and the university reduced the course to a duration of 9 weeks. This left 5 weeks to deliver the course online and 2 weeks to complete the assessment and final examinations.

To ensure that the remaining course objectives were met, 10 faculty members collaborated to identify the most crucial topics in the fifth-year undergraduate medical curriculum. They also decided whether the educational activity should be delivered asynchronously or synchronously. These faculty members identified 11 asynchronous lectures and 17 synchronous CBD to be delivered online over 5 weeks by 50 pediatric faculty members according to their area of interest or subspecialty.

### Video conferencing session administration

For 5 weeks, each instructor facilitated 1 or 2 CBD sessions weekly to cover 17 CBD topics. Each session was allocated a 2-h duration and involved a set of 1 or 2 groups of students (10–12 students per group). The WVC options were Blackboard Ultra and Zoom. Because Blackboard Ultra is the learning management system (LMS) platform used by the university for course delivery, it faced high pressure during this period since most university instructors were utilizing it. After piloting both platforms, the instructors reported easier navigation and fewer interruptions, connection dropouts, and audio-visual problems using Zoom.

Zoom is an innovative meeting company that unifies cloud WVC, simple web meetings, and group collaboration in one easy-to-use platform. It has been one of the most extensively used tools for facilitating ongoing synchronous educational activities during the COVID-19 pandemic [24, 25]. Furthermore, it allows wireless screen and whiteboard sharing, has an interactive chat room, and is free for up to 40 min. Thus, it is an optimal choice for facilitating synchronous teaching activities to provide an optimal learning experience with enhanced student and instructor engagement [26].

In preparation for the CBD sessions, instructors received the CBD learning outcomes and the list of students participating in the discussion. Additionally, a full orientation on using Zoom was provided for all academic pediatric faculty members through a half-day workshop. After the workshop, an e-mail link was sent to both students and faculty members with a demonstrative video recorded by one of the pediatric faculty members providing simple explanations on utilizing Zoom in Arabic.

### Students’ evaluation of educational quality

The reduced version of the Students’ Evaluation of Educational Quality (SEEQ) was chosen as the survey tool to evaluate the teaching quality of the WVC CBD sessions. The SEEQ was developed by Marsh in 1987 to assess the level of student satisfaction with teacher effectiveness to improve teaching quality [27]. The reduced SEEQ has been internationally validated for research studies [28]. It has a robust quality, excellent reliability and internal consistency, and reasonable validity, and has the flexibility to fit individual teaching contexts. It is useful for rapidly acquiring data and minimizing the risks of item non-response, and it has been extensively utilized in many courses worldwide at both the graduate and undergraduate levels [29, 30]. The SEEQ questionnaire examines nine characteristics of effective learning: learning, individual rapport, enthusiasm, examinations, organization, breadth, group interaction, assignments, and overall rating.

### Data collection

Following an initial pilot questionnaire completed by one group of 10 students and reviewed by three pediatric faculty members, the questionnaire categories were tailored to be more relevant to the WVC CBD sessions. We eliminated 2 questions from the original reduced SEEQ questionnaire, one under the category of enthusiasm related to using humour, and the second under the category of individual rapport related to the instructor being friendly toward individual students. Moreover, we added 2 technology questions relevant to the use of WVC. The final questionnaire contained the following 21 items: 1 question on the assigned student group; 2 questions about the CBD session (topic, date, and time); 14 reduced SEEQ factors focusing on learning (Q4–Q7), enthusiasm (Q8–Q9), organization (Q10–Q12), group interaction (Q13–Q15), and individual rapport (Q16–Q17); we added 2 questions relevant to WVC technology usage (faculty creativity and audio/visual technical issues); 1 question about student satisfaction with the CBD session quality; and 1 open-ended question that allowed students to comment on their experience. A 5-point Likert scale ranging from 1 (strongly disagree) to 5 (strongly agree) was used.

After approval from the Research Ethics Committee at King Abdulaziz University Hospital, an attached invitation letter was sent via weblink explaining the purpose of the study and students were asked to complete the survey for one CBD on a weekly bases for 5 weeks duration, and to rate the degree to which they agreed or disagreed with each item on the survey. The SurveyMonkey was sent over the course of 5 weeks to all group leaders to be distributed among their fellow students. The students’ participation was voluntary and anonymous, and they were able to withdraw at any time.

### Statistical analysis

Data were collected using SurveyMonkey and then analyzed using Statistical Package for Social Science (SPSS) version 22.0 to determine the means and standard deviations of the questionnaire items. The analysis relied on having no group differences. The mean for each of the items was compared between the high-satisfaction group (HSG) and low-satisfaction group (LSG) with a 95% confidence interval (CI) using independent sample t-tests. A student was assigned to the HSG if the overall CBD satisfaction response rated as agree or strongly agree on the 5-point Likert scale or to the LSG if he rated the response as strongly disagree, disagree, or neutral. Pearson’s correlation coefficient was used to assess the correlations between student satisfaction and technology usage.

## Results

A total of 662 male student responses were received (an overall response rate of 82%). Table 1 displays the descriptive analysis of the of reduced SEEQ responses by degree of overall Student Satisfaction. As shown, students rated highest mean score in the items under the category of teaching presence “students were encouraged to participate” and “faculty members’ explanations were clear” with a mean score of 4.23 (± 0.78), and 4.20 (± 0.85) respectively. Under the technology category “During this CBD session, there were audio or visual technical issues” and “the faculty member creatively used the whiteboard, chat room, and videos” students had the lowest mean scores of 3.95 (± 0.99) and 2.82 (± 1.35), respectively.

**Table 1:** Descriptive Statistics of Responses to the Reduced SEEQ by Degree of Overall Student Satisfaction

SEEQ item	Overall Mean (SD), *N* = 662	HSG Mean (SD), *N* = 543	LSG Mean (SD), *N* = 119	MD (95% CI)	*p* - Value
*Learning					
4 I have found this CBD session intellectually challenging and stimulating.	4.08 (0.863)	4.30 (0.629)	3.08 (1.062)	1.218 (1.074–1.362)	<0.001
5 I have learned and understood the subject materials of this CBD.	4.12 (0.836)	4.35 (0.588)	3.09 (1.008)	1.257 (1.122–1.393)	<0.001
6 My interest and motivation in learning have increased as a consequence of this CBD session.	4.04 (0.934)	4.29 (0.684)	2.89 (1.056)	1.398 (1.247–1.550)	<0.001
7 The faculty member covered the stated objectives for this CBD session.	4.162 (0.821)	4.354 (0.654)	3.286 (0.931)	1.067 (0.926–1.209)	<0.001
*Enthusiasm					
8 The faculty member was enthusiastic (excited) about teaching this CBD session.	4.17 (0.852)	4.38 (0.620)	3.18 (1.057)	1.196 (1.054–1.339)	<0.001
9 The faculty member was dynamic (style of presentation holds your interest) and energetic during this CBD session.	4.08 (0.949)	4.35 (0.659)	2.86 (1.099)	1.493 (1.342–1.643)	<0.001
*Organization					
10 The faculty member’s explanations were clear.	4.20 (0.851)	4.43 (0.603)	3.15 (1.022)	1.276 (1.138–1.414)	<0.001
11 The CBD material was well prepared and carefully explained.	4.10 (0.908)	4.35 (0.624)	2.97 (1.119)	1.380 (1.233–1.526)	<0.001
12 The faculty member gave a CBD session that facilitated taking notes.	4.06 (0.917)	3.00 (1.112)	3.00 (1.112)	1.289 (1.136–1.443)	<0.001
*Group interaction					
13 Students were encouraged to participate in this CBD session.	4.23 (0.781)	4.40 (0.586)	3.47 (1.064)	0.925 (0.787–1.064)	<0.001
14 Students were invited to share their ideas and knowledge.	4.19 (0.829)	4.37 (0.617)	3.34 (1.108)	1.026 (0.881–1.171)	<0.001
15 Students were encouraged to ask questions and were given meaningful answers.	4.18 (0.819)	4.35 (0.642)	3.43 (1.078)	0.919 (0.773–1.066)	<0.001
*Individual rapport					
16 The faculty member made the students feel welcome in seeking help/advice regarding learning challenges during COVID-19.	4.04 (0.944)	4.26 (0.749)	3.06 (1.107)	1.199 (1.035–1.363)	<0.001
17 The faculty member had a genuine (sincere) interest in students.	4.08 (0.874)	4.29 (0.674)	3.13 (1.030)	1.167 (1.018–1.316)	<0.001
*Technology					
18 The faculty member was creative (used whiteboard, chat room, videos, web materials, etc.).	3.95 (0.996)	4.15 (0.838)	3.03 (1.134)	1.121 (0.942–1.300)	<0.001
19 During this CBD session, there were technical issues (audio/visual).	2.82 (1.355)	2.85 (1.389)	2.67 (1.180)	0.177 (-0.092–0.446)	0.198
*Satisfaction					
20 Overall, I was highly satisfied with this CBD session.	4.10 (0.883)	4.44 (0.496)	2.59 (0.643)	1.848 (1.744–1.953)	<0.001
**21 Do you have any comments ?** “Dr. X was very enthusiastic during teaching. It was a very beneficial CBD”. “One of the most beneficial CBDs I took this year”. “I couldn’t ask all my questions, Dr. X immediately logged off the app and didn’t wait to see if we had questions” “Was a good experience with Dr. X but she took so long around 3hours, overall she was a really good tutor” “Dr. X was very helpful and encouraging”. “Dr. X is a good example of how a doctor should encourage her students to be strong and to study hard. She has a good method of delivering the information to the students in an easy way”.					

*SD* Standard deviation, *N* Number of students, *HSG* High satisfaction group, *LSG* Low satisfaction group, *MD* Mean difference *CI* Confidence interval.

Under the learning category, which was equivalent to cognitive presence, the majority of students agreed that the sessions were intellectually challenging, and that they understood the subject materials, with a mean score of 4.08 (± 0.863) and 4.12 (± 0.836), respectively. Moreover, under the category of group interaction and individual rapport, which reflected both teaching and social presence, the students agreed that the instructor invited them to share knowledge and had a genuine interest, with a mean score of 4.19 (±0.829) and 4.08 (± 0.874), respectively.

Furthermore, 82% of the students were in the HSG versus 18% in the LSG, with a statistically significant difference in the mean scores in all categories *(p* < 0.001) except for the item “facing technical issues” (*p* = 0.198). A statistically significant difference was found in student satisfaction between the HSG and the LSG, with a mean score difference of 1.848 (95% CI, 1.744–1.953; *p* < 0.001). No statistically significant correlation was found between student satisfaction and technical issues (*r* = 0.037, *p* = 0.003).

## Discussion

The COVID-19 pandemic compelled the educational system to enter a new era of online learning. This study investigated the novel exposure to distance education for both students and faculty members. To the best of the authors’ knowledge, this was the first study to explore student satisfaction with pediatric WVC CBD sessions’ teaching quality. It provided insight into the strengths and weaknesses of student satisfaction with the teaching quality of WVC CBD sessions. Students scored the highest mean under the category of teaching presence while scored lowest under the category of technology. This indicated that students were satisfied with teaching presence despite faced challenges with technical issues, and that faculty members could improve their WVC navigation. All students had self-acess to smart devices and internet connection. Infrastructure support was provided for both students and faculty members when Blackboard platform was utilized including navigation and technical support, but not with Zoom. Of the participating students, 82% had a high satisfaction rate in all of the categories on the SEEQ questionnaire. The students’ high satisfaction with the sessions’ quality was not surprising considering the sessions’ compatibility with the COI framework. Conrad in his literature review article explored the impact of video technology on each of the three elements of the COI framework. He points out that WVC helps with building a COI framework by establishing social presence for both the instructor and the student, engaging learners and learner-learner interactivity, increases teaching presence by allowing robust feedback and conveying of emotions to the students, and enhances cognitive presence by allowing a flipped classroom model of teaching, which encourages critical thinking and processing of information [31].

The results supported those of other studies that showed that instructor presence and an interactive teaching style were significant determinants of student satisfaction and the effectiveness of online learning environments [32, 33]. Additionally, the findings of the present study were in line with Duygu’s study, which revealed a positive student perception of the teaching content, effectiveness, interaction, and idea-sharing of WVC despite experiencing technical problems related to sound quality and connection [34]. Moreover, the findings were consistent with a study by Doggett [16] that showed a positive student experience with the instructor’s use of WVC technology and teaching skills despite students’ perception that WVC technology was a barrier to interacting with the instructor. Several other studies during the COVID-19 pandemic similarly highlighted the effectiveness of WVC for delivering online teaching, engaging students, and establishing a sense of community and social presence, thereby promoting a positive student experience despite visual and audio issues [35–37].

The study found that teaching effectiveness and quality relied on teaching, cognitive, and social presence and not on technology. However, technology remains an important platform that supports teachers’ educational activities. Additionally, this study illuminated the importance of satisfying the growing demands for online education while maintaining a worthwhile student learning experience.

Therefore, standards should be established for a blended-learning pediatric course that combines traditional clinical skills, face-to-face training, and innovative educational methods. Faculty development workshops and training on distance education and teaching skills and competencies should be organized, and training on navigating WVC platforms should be provided for both students and faculty members. Furthermore, set expectations should be established for the WVC netiquette system, and technical issues should be addressed by utilizing the online technical support system. Additionally, the effect of such implementations on future pediatric course quality and student and faculty satisfaction should be monitored. Finally, this study has the potential to enhance WVC teaching effectiveness and enrich online course delivery in other medical and healthcare fields.

## Conclusions

WVC teaching had an overall positive outcome on student satisfaction, and teaching quality relied on teaching, cognitive, and social presence rather than technology. However, technology remains an important platform that supports teachers’ educational activities. Thus, implementing a blended pediatric course to augment future course delivery is optimal.

### Limitations

The study had certain limitations. First, it was conducted on one group of male students rotating in pediatrics in a single center. Therefore, inferences cannot be made about other courses or faculty members for which the education culture and environment may be different. Second, the survey responses may have been limited by inaccuracy and subjected to recall bias, since some students did not complete the survey immediately after the CBD. Third, different faculty members facilitated the CBD sessions and information on previous online course teaching was not collected. Therefore, not all students were exposed to the same orientation (such as online netiquette system and expectations) or online teaching style, skill or competency. Fourth, the study did not elaborate on student retention or success rates since the students’ performance evaluations were set as pass or fail grades, and 98% of the students completed and passed the course successfully. However, this rate of completion reflected positively on the success of the teaching quality of the WVC CBD sessions.

## Data Availability

The adjusted reduced SEEQ questionnaire used during the current study are available from the corresponding author on reasonable request.

## Abbreviations

Covid-19: Coronavirus disease 2019
LMS: Learning management system
WVC: Web videoconferencing
SEEQ: Students’ Evaluation of Educational Quality
CBD: Case based discussion
VC: Videoconferencing
COI: Community of inquiry framework
DPBL: Digital problem-based learning
PBL: Problem based learning
